# Supplementary material for: Neural cells are susceptible to historic and recently emerged Oropouche virus strains
Source: PLoS Pathog. 2026 Feb 9;22(2):e1013933. doi: 10.1371/journal.ppat.1013933 (PMC12904570; doi:10.1371/journal.ppat.1013933)
Supplement: Attachment — Submitted filename: Response to Reviewers.docx [file ppat.1013933.s002.docx]

We appreciate the reviewer’s thoughtful and thorough analysis of our manuscript. We have addressed their comments with our responses below (in blue) and the appropriate changes made to the text and figures.

Reviewer #1: Line 326. While the BeAn19991 strain was derived from a reverse genetics system, the absence of detailing the passage history for this strain and the associated strengths and weaknesses that accompanies the passage history must be described. The citation for this reverse genetics system is included in the methods (Tilson et al. 2015; PMID: 26699638) and describes the passage history of the BeAn19991 strain. We added some clarification in the discussion on how the passage history could influence our results.

The experimental design is really unclear why so many MOIs were used with just one OROV strain, and the recent isolates weren’t used for comparison in any of the immortalized neurons, microglia and astrocytes. This would be very informative information given that most research is focused around the recent isolates and not the 1960 isolate. The need for these comparisons are also supported by the differences observed between the ancestral and contemporary isolates in rat cortical neurons and BSC gene expression studies. We agree with this comment. These studies began prior to the contemporary outbreak of OROV. The MOI limitations we had were due to low titer of the stock virus combined with the confluency of the particular cell type being analyzed. The highest MOI possible were used within his study, and we tried to be clear about which MOI was used for each figure panel.

Reviewer #2: Major Comments:
1. Fig. 1G, H, I: the images lack sufficent resolution (e.g. beta III Tubulin staining is clustered, Iba1 staining is extremely faint, Phalloidin staining seems very odd and variable). The authors should I. Add scale bar We added scale bars to all images II. Incorporate higher magnification, better quality images Images were re-exported and converted to TIFF files at 300 dpi. III. Add IFA quantification of viruses in different cultures. We agree that this would be interesting to have however the student working on this project is no longer available to do it. IV. Stain astrocytes with classical markers like GFAP, GLAST etc. We added images for GFAP staining of the mouse astrocytes (C8-D1A). We attempted to stain the human hTERT-astrocytes with several GFAP clones which did not work.
2. The authors didn’t report any cytopathic effects (CPE) even after long endpoints e.g. 4 days. Is there no visible CPE even with a high MOI inoculum? We did not specifically comment on CPE but there is loss of cell integrity over time in the cultures through 72 hrs. It is difficult to compare across different cell types and cultures because most of the cells used here do not grow in even monolayers. If so, the authors should infect the cultures with more virulent lines like BR-2015-2023, to evaluate the cause of neurovirulence and lethality in emergent isolates. The two emergent strains we used (AM0088 and 240023) are both strains within the BR-2015-2023 lineage. The strain AM0088 was isolated from Brazil (see Scachetti, 2025) and the 240023 isolate is a novel 2022-2024 strain isolated from a febrile patient from Cuba (see Payne, 2025).
3. In Fig. 2D, why do the neuronal primary cultures have only a few infected cells, even though neurons lack strong immune response and should be highly permissible to neuroviruses like OROV? The authors should use higher inoculum dose and emergent lethal isolates to test this. The neuronal cultures have may infected cells by 48 hpi. To provide clarity, we increased the size these images to better visualize the viral antigen staining over time. We also added the single-channel image for OROV antigen staining to each timepoint to allow for better visualization of infection over time (see revised Fig 2B and 2D).
4. In Fig. 3A, why are the kinetics of the new strains slower even though these are more emergent with neurological symptoms? This is a good question, and when we began these studies, we hypothesized that the emergent strains would have faster replication kinetics due to their increased neurological sequelae. Very early reports (See Scachetti MedRXIV preprint from July 30, 2024 Fig 4; subsequently published in Fig 3 in Lancet Inf Dis; see also Gunter et al preprint Fig 3 <http://biorxiv.org/lookup/doi/10.1101/2025.08.02.668287>) indicated that perhaps the emergent strains replicated faster, at least in Vero cells. In HTR8, BeWo and JEG3 cells there appeared to be comparable replication kinetics between BeAn19991 and 240023. However, our study here, as well another preprint (doi: <https://doi.org/10.1101/2025.08.13.669874>) suggest that BeAn19991 replicates more robustly than 2024 isolates in neural origin cells. In the cell types tested here, we found that the historical BeAn19991 strain replicated to higher titers than the emergent strains. There could be many reasons for this: the BeAn19991 strain is rodent-adapted and/or the emergent strains are more neuroinvasive, which is not a characteristic assessed in this paper. Previous studies have demonstrated orthobunyavirus specific differences in neuroinvasion (Evans, 2022), which could explain the current clinical data if there is enhanced neuroinvasive capacity for the emergent strains that does not necessarily correlate with in vitro replicative capacity. Additional epidemiologic evidence is needed to affirm the relationship between clinical symptoms in historical and emergent strains of OROV.

Also, in Fig. 3B, 240023, the staining of beta III Tubulin seems more clustered, probably directing virus-induced trafficking/ modulation of host factors. The authors should further investigate such differential characteristics between historic and emergent strains. In addition, these observations should be correlated with CPE in a later time point/ higher MOI infection. We do agree that there are visual differences in the Beta III tubulin staining for both 240023 and AM0088 (when compared to BeAn19991) which could indicate host modulation that will be explored in future studies.
5. In Fig. 4, do the authors have any thoughts/explanation as to why the emergent neurovirulent strains produce similar PFUs/ml, compared to the historical strain? See above, point 4. Also, the Panel 4B images look out-of-focus; the authors should provide clearer maximum-intensity projection images for slice cultures. This has been fixed with the higher-quality images provided upon re-submission. The authors should parallelly stain with neuronal/ NPC markers to demonstrate cell-specific localization with virus (in a higher-magnification). We hope to explore neurotropism in whole brain sections in the future using in vivo studies. In Panel C., the historical strain induces more IFNB compared to others, are the emergent strains capable of interfering the neuronal immune response, inducing neurovirulence? Possibly! If these emergent strains have enhanced neuroinvasion, a greater interference with interferon responses in the periphery could explain this difference.
6. The authors should compare the neuro-invasiveness in animal models to compare strain-dependent pathologies in vivo. This would be an excellent next step for understanding the neuropathogenesis of these strains in vivo.

Reviewer #3: This paper represents a well-executed characterization of growth characteristics of OROV strains in a variety of permissive cell lines, primary cells, and ex vivo rat brain slice cultures. Overall, the growth kinetics are very similar between OROV-BeAn19991 and the two more recent isolates. However, the authors bury the lead and the most interesting difference between these strains appears to be the increased inflammasome induction by OROV-AM0088, which may have important consequences in the increased findings of OROV-associated neurologic disease. This finding is not highlighted in the abstract and comprises the tail end of the discussion—it should be a more prominent focus of the manuscript. This is an excellent observation which we did not follow up on for this set of studies. A comparison of inflammasome activation between historical and emergent strains, and how this may perhaps contribute to the increased incidence of disease, would be an interesting next step for the student that hopefully takes over this project.
2. The reverse genetics system could be used to determine which sequence polymorphisms are associated with NLRP3 induction. Or, the authors could generate genome reassortants to determine which segment is influencing NLRP3 induction. We completely agree that this is a logical next step.

**Part III – Minor Issues: Editorial and Data Presentation Modifications**
Please use this section for editorial suggestions as well as relatively minor modifications of existing data that would enhance clarity.

Reviewer #1: Viruses section. Please provide some of the metadata for these viruses within the manuscript. Host, year of isolation, passage history, etc? This information has been added to the discussion.
Line 52. OROV is the acronym for Oropouche virus. Please correct. Fixed.
Lines 41-42 “including alarming incidences of neurological issues in confirmed patients.” is unclear. Please be more specific. Done
Lines 58-59 “…febrile illness with severe headache, chills, …” should be “…febrile illness characterized by headache, chills, …”. Done
Line 335. …Was kindly provided… Done
Lines 336-337. “To generate reverse genetics” is a very unclear phrase. Please rephrase for clarity. Clarified
Table 2 is missing the NSs comparison between BeAn19991 and 240023. Is this protein absent in the 240023 strain. Clarified
Lines 404-405. There was no attempt of description of the methods used to determine or confirm the differentiation into neurons. This should be performed for any cell type that was differentiated. These primary cells were not differentiated in vitro. They are isolated from animals and cultured ex vivo.
Figures 1 and 2 present results of infectious titers yet the figure legend and methods describe the use of RT-qPCR. No description is provided if they performed a standardization procedure to be able to graph RNA loads as pfu/ml. This is needed to assess the rigor of estimating your equivalents. Added information to the methods on the development and parameters of the RT-qPCR.
Line 146. “were” should be “where” Fixed
Line 167. Give the rational for using a high titer (10^5 pfu) for infecting the BSCs. Added
Lines 169-170. With the exception of OROV-240023, I’m not convinced of replication for the other 2 strains in BSCs since there is no or very little increase in viral titers. The authors don’t necessarily rule persistence of the virus over time in the culture. It would be good to know what the 0hr timepoint titer would be, to see if there was an increase between the 0hr and 24 hr time points. We agree that the increase in viral titers over time is modest at best, especially when compared to our previous study in BSCs using RVFV (see PMID: 38546100). We added some potential reasons for these lower titers over time in the BSCs within the discussion section and emphasized the increase in viral antigen staining observed using whole-slice immunofluorescent microscopy.
Lines 204-210. This is a valuable discussion point to compare the sequence identities. However the authors don’t emphasize any particular discussion point, but instead restate some of the results. What does this sequence similarity mean in the context of the proposed altered pathogenesis of the recent emergent strains? Or is it important to compare sequence similarity among all the complete genomes among the current OROV sequences available to help support your point. This sort of general analysis could be cited if already done, or at least serve to increase citations of this work. We modified this section to include citations of previous work.
Line 222. This is unclear and needs rephrasing
Give some rational as to why the proliferating neurons (N2a and SHSY-5Y) were not differentiated but used as is for a proxy for neurons. We clarified this and added rationale to discussion.
Lines 256-258. I think these comparisons should be included in this study. Maybe do a single strain with a reduced number of MOIs for comparison if needed. This comment was noted above as well. We agree that this would add useful information to our understanding of OROV infection and neurogenesis, but these studies would require extensive optimization as we are not currently equipped to perform them.

Figure 4 and lines 289-296. It’s not a good comparison. If the gene expression studies in mock were done at all 3 time points, then shouldn’t there be 6 points and not 4 as seen in the graph? We used 6 mock slices as controls for viral titer (not n=2 as previously listed) and a total of 5 slices for gene expression across the two experiments. The figure legend has been updated to represent this.

Also, is there another measure of cellular health you can use to rule out the fact that the increase IL-1B is result of virus cytopathic effects and not natural cell death during maintenance, which you will see in mock controls. We did not perform studies related to cellular health for this project. We previously demonstrated that there is an inflammatory response in culture after plating BSCs, which resolves over time (see PMID: 38546100). The mock slices used in this study serve as the comparison for the amount of IL-1b being released from these slices, even in extended culture. Additional cellular health assays, as used in our original publication, will be used in the future.

Reviewer #2: Minor Comments:
1. The authors should explain more about the difference between the historic and emergent strains of OROV (mainly on infectious capability, neurotropism, immune responses) in the introduction as that’s the main theme of this manuscript. Given the limited number of in vitro studies (none on neurotropism or immune responses) on the emergent viruses, we focused on the epidemiological differences in the introduction as it better highlights the neurological disease associated with Oropouche fever. We expanded more on the current in vitro work across the strains in the discussion.

2. Do any prior studies demonstrate differences/ comparative pathologies between isolates from any severe infectious cases with neurological symptoms, any comparable data on those? Not to our knowledge.
3. Fig. 1A-F should show different doses in different colors, the different shades of green are not easily distinguished. We modified the figure to have different points and connecting lines to make the figure more easily readable.

4. In Fig. 1 C-D and G, compared to the neuronal lines, why is prominent virus infection observed by IFA that's not reflected in titer (i.e. reduced in microglia)? It is possible that, while antigen staining is robust, the infected microglia are not producing as much virus and viral RNA that would be reflected in the growth curves.

5. Do the astrocytes maintain low plaques production to harbor the virus for a longer time i.e. act as a viral reservoir in neuroinfection? This is an interesting observation. Prior studies demonstrate that astrocytes are not infected by OROV, so our low levels of infection could demonstrate that astrocytes do restrict OROV infection in some capacity which we are unable to recapitulate in a monoculture system.
6. In Fig. 2, the mocks should be put in left, followed by initial-to-distant time post infection. Also, Panel B and D lacks clarity (D: Mock b III Tubulin image is not focused), thus should be added with higher magnification images. Mocks have been moved. The beta III tubulin in these cultures is not completely in focus, as these cultures tend to form clumps, resulting in some areas that are unfocused in each field of view.
7. The authors should use L-Glutamine instead of I-Glutamine, in case they have not used something different. Fixed!
8. The authors should mention the plasmids used for reverse genetics. This is an interesting system, so they may think of describing it in more detail. The methods used for rescuing the recombinant AM0088 are described in Tilston 2015, and the plasmids were the same here (pTVF). We clarified this in the methods section.
9. The authors should use more neurovirulent strains like BR-2015-2023 (that they mention in discussion) to evaluate mechanisms of increased neuropathogenesis in emergent strains. Do the authors predict any potential difference between CNS entry mechanisms? The two emergent strains we use here are both isolates from the BR-2015-2023 lineage (~98% sequence identity per NCBI blast). We do suspect there may be differences in neuroinvasive capacity, which is not demonstrated in the current manuscript.
10. Considering the relatively modest amount of data presented, the discussion is extremely long and somewhat rambling. Would suggest presenting a more succinct and focused discussion. Thank you for this note. We significantly shortened the discussion to provide only the most important information to summarize the context of our work.

Reviewer #3: Non
